# Impact of Dark Triad personality traits on COVID-19 vaccination uptake and prevention efforts: insights from the European Covid Survey (ECOS)

**DOI:** 10.1186/s12889-025-22471-3

**Published:** 2025-04-10

**Authors:** Sophia Bock, Sebastian Neumann-Böhme, Petra Steinorth

**Affiliations:** 1 Institute for Risk Management and Insurance, University of Hamburg, Moorweidenstraße 18, 20148 Hamburg, Germany; 2 Hamburg Center for Health Economics, University of Hamburg, Esplanade 36, 20354 Hamburg, Germany

**Keywords:** Public health, Demand for vaccines, Decision-making under risk, Dark Triad personality traits, SARS-CoV-2, Preventive behavior

## Abstract

**Background:**

Even though the COVID-19 vaccination roll-out in general can be considered as one of the most successful public health campaigns in the history of medicine, general vaccination hesitancy has remained an issue of concern throughout the world. We add to a deeper understanding of vaccination hesitancy by identifying what drives primary vaccination and booster uptake, as well as adherence to simple preventive measures such as physical distancing by investigating the role of Dark Triad personality traits, i.e. Machiavellianism, narcissism, and psychopathy.

**Methods:**

We investigate data from Germany and the United Kingdom from the European Covid Survey which was collected from 23 December 2021 to 10 January 2022. Logit regressions and random effects regressions were performed to study the effect of dark personality traits on COVID-19-related prevention.

**Results:**

We find a statistically significant association between Dark Triad personality traits and prevention efforts, primary vaccinations, and booster uptake against COVID-19. Specifically, individuals scoring high in psychopathy are associated with a lower likelihood of having received primary immunization. The marginal effect amounts to 3.31%-points. High narcissistic personality traits are correlated with a substantially higher likelihood (4.52%-points) to refuse booster shots after having received the primary vaccinations. Dark Triad personality traits may be relevant factors associated with vaccine-related decision-making. In addition, individuals with higher psychopathic tendencies report significantly lower engagement in other simple preventive behavior, while higher scores in narcissism are associated with higher reported adherence to simple preventive measures.

**Conclusions:**

Our findings highlight the crucial role that personality plays in pandemic-related prevention. Policymakers, health professionals, and those in charge of health messaging may take these factors into account when devising communication strategies to improve the vaccination uptake and adherence to preventive behaviors. Future pandemics and public health crises would benefit from targeted, nuanced approaches to public health messaging to promote greater public adherence and public health.

**Supplementary Information:**

The online version contains supplementary material available at 10.1186/s12889-025-22471-3.

## Introduction

Vaccination hesitancy is a global phenomenon where a substantial part of the population is not getting vaccinated despite having access to the vaccine. Even though the COVID-19 vaccination roll-out in general can be considered as one of the most successful public health campaigns in the history of medicine, general vaccination hesitancy has remained an issue of concern throughout the world [1, 2], even within high risk groups such as pregnant women [3, 4]. The World Health Organization reports about 5.2 billion of the overall 7.8 billion global population have received full primary immunization for COVID-19 as of the end of the year 2023.

The decision to get a vaccination is a prevention decision, which requires an investment upfront (the actual vaccination and potential side effects thereafter) in the expectation of the long-term benefit of immunity as theoretically discussed by Courbage and Peter [5]. The fear of future side effects is one of the highest ranked reasons for hesitating to get vaccinated [6, 7]. Science has provided conclusive evidence that (public) health benefits by far exceed adverse outcomes from vaccinations. Nevertheless, individual preferences and behavioral biases may imply that individuals prefer not to get vaccinated or take protective action, as suggested by Campos-Mercade et al. [8], Müller and Rau [9], Zettler et al. [10] or Ścigała et al. [11]. In addition to the initial COVID-19 vaccination hesitancy among some groups within the population, also a sizeable number of individuals opted out from booster shots who had received primary vaccinations. The necessity for regular booster shots only became evident over time after promising results on vaccine efficiency at the end of 2021 [12]. New variants emerged and evidence showed that booster shots were required for long-term prevention as antibodies reduce after a couple of months.

Individuals can also take (simple) personal preventive measures such as reducing contacts to others or using hand sanitizer as discussed in Smith et al. [13]. Stoddard et al. [14] use a game-theoretic approach to explore noncompliance with non-pharmaceutical and biomedical measures during a pandemic and show that noncompliance can be an individually optimal choice in terms of cost-benefit under a broad set of conditions. Agusto et al. [15] study (rational) behavior of susceptible individuals in the context of social distancing to explain the second wave of the COVID-19 pandemic. Beyond previous results, Bughin et al. [16] highlight the trade-off between vaccination and non-pharmaceutical interventions such as social distancing as individuals balance these two types of prevention to protect against COVID-19.

Furthermore, Sabat et al. [17] find evidence for vaccination hesitancy as the most unstable vaccine intention with on average 42% of ever-hesitant individuals remaining hesitant in future waves. Understanding the dynamics and determinants of vaccination efforts is crucial for the success of targeted campaigns for future health crisis.

We contribute to a deeper understanding of vaccination hesitancy by examining factors associated with vaccination uptake, specifically differentiating between primary vaccination and booster uptake, as well as adherence to WHO recommendations on simple preventive behavior by investigating the role of *Dark Triad* (SD3) personality traits, i.e. Machiavellianism, narcissism, and psychopathy, as introduced by Paulhus and Williams [18], in these three prevention decisions.

The Dark Triad personality traits are relevant for identifying behaviors and power structures in groups [19], but also with COVID-19-related vaccination hesitancy [20] and for tailoring public health messages to increase adherence [21]. Individuals who score high in *Machiavellianism* are self-interested, ruthless, and cynical manipulators, who deceive for material gain, e.g. by misreporting [22]. Individuals with high *narcissism* have a sense of grandiosity and entitlement. High scores in *psychopathy* lead to antisocial behavior, impulsivity, and thrill-seeking. Psychopathy is associated with less compliant behavior as well as social distancing [23], and greater beliefs in COVID-19-related conspiracy theories [24]. However, other research suggests that a major health crisis itself can influence the stability of social preferences and behavior, as discussed by Shachat, Walker, and Wei [25], Li et al. [26] or Lohmann et al. [27]. While for example Lohmann et al. [27] find that high exposures to COVID-19 causes lead to an increase in anti-social behavior, mental health support in the early stages of a crisis may be critical.

We contribute to the existing literature on the demand for vaccines, Dark Triad personality traits, and behavior during the COVID-19 pandemic by specifically analyzing the propensity for booster shots and other simple preventive measures in addition to primary vaccination. These preventive behaviors are recommended by the WHO as socially preventive and responsible behavior [28]. We do find distinct associations between malevolent personality traits and these three different dimensions of prevention during the COVID-19 health crisis. Our analysis suggests that individuals with narcissistic personality traits are associated with a higher likelihood of reporting to refuse booster shots after having received the primary vaccinations, while individuals scoring high in psychopathy are significantly less likely to report to have received primary immunization. Individuals with higher psychopathic tendencies exhibit lower adherence to other simple preventive behavior, while higher scores in narcissism are associated with higher reported adherence.

We also contribute to the literature that investigates potential barriers and determinants of (general) vaccination uptake and prevention, ranging from socio-demographic factors, social norms and beliefs, and previous experiences [29–31], people’s level of religiosity [32, 33] or cognitive factors such as susceptibility and severity [34]. At the aggregate level, the literature emphasizes the role of the trustworthiness of public organizations or institutions [35–37], cost of vaccines [38], and anticipated consequences of non-pharmaceutical interventions [39]. Furthermore, Böhm and Betsch [40] and Böhm, Betsch, and Korn [41] find that pro-social concerns increase vaccination uptake. In addition, findings suggest that risk-averse individuals are more likely to be vaccinated as shown by Benzion, and Shahrabani [42], among others.

Our analysis is based on the longitudinal European COvid Survey (ECOS). The study was originally conducted to analyze people’s attitudes and concerns regarding the novel coronavirus SARS-CoV-2, including items from the COVID-19 Snapshot Monitoring Project (WHO). In particular, previous publications analyzed probable depression and anxiety during the pandemic [43], the uptake of the WHO recommendations [44], the link between altruism and pro-social pandemic behavior [45], people’s perceptions about the policy responses [46] or vaccination uptake in general [6].

After this introduction, the paper is structured as follows: We first introduce background of the Dark Triad personality and discuss potential effects on COVID-19-related preventive behavior. Methods section gives an overview of the data and methods we use in our analysis. We then show results from our analysis in Results section. Discussion section offers some discussion and policy implications are addressed. In Conclusions section, concluding thoughts are presented.

## Dark Triad personality and COVID-19-related vaccination and other preventive behavior

The Dark Triad personality consists of the three conceptually distinct subclinical personality traits Machiavellianism, narcissism, and psychopathy [18]. While these constructs are of overlapping nature^1^ the literature provides evidence of distinctive differences [18, 47–49]. With regard to the COVID-19 pandemic, some literature studies a potential differential impact of the three personality constructs on health-behavior endorsement and the appeal of public health messages [21] or psychological consequences [50, 51].

In the following, we discuss how the individual personality traits of the Dark Triad potentially affect primary immunization, booster uptake, and other simple preventive behavior. Table 1 summarizes the results of the discussion of the following three sections.

**Table 1:** Predictions of dark personality traits and COVID-19-related behavior

	Primary Immunization	Booster Uptake	Preventive Behavior
Machiavellianism	+/-	+/-	+
Narcissism	+/-	-	+
Psychopathy	-	-	-

“+” indicates that a positive effect is expected, “-” negative effect is expected, and “+/-” no difference to the rest of the sample is expected

We do not expect individuals with high Machiavellian personality traits to differ from the rest of the sample with respect to the primary vaccination and booster shots, but to be more likely to adhere to preventive measures as we discuss below. We do not expect narcissistic individuals to be more likely primary immunized than the rest of the sample, but to be more likely to opt out from the booster shot. In addition, we expect individuals with high narcissistic personality traits to be more likely to report adherence to preventive measures as we explain in Narcissism section. For individuals with high psychopathic tendencies, we expect fewer vaccinations, less booster uptake, and less preventive behavior.

### Machiavellianism

*Machiavellians* have the tendency to be manipulating, calculating, and ruthless individuals with little regard for others [18]. Generally, Machiavellians carefully consider the odds of their behavior and often use antisocial methods to achieve their goals, justified through rationalizations [52]. While these tendencies can lead to useful social outcomes under certain circumstances, Machiavellians can use the pandemic to exploit and manipulate others to serve their self-beneficial goals [48]. In sum, Machiavellianism comprises several different core themes and can be seen as a multidimensional construct [53].

As the primary immunizations and booster shots are considered safe and are recommended by health authorities, we would not expect Machiavellians to be more likely to opt out from vaccinations than individuals with less pronounced Machiavellian personality. This may hold true even though concerns about protecting others from infection can be considered a lower priority for Machiavellians. With respect to other simple preventive behavior, Machiavellians can be less affected by pandemic fatigue^2^ due to their rationality and lower individual costs compared to vaccination and therefore adhere more consistently to preventive measures if they consider them effective. In addition, over-reporting of adherence can be seen as a reputation building tactic as Machiavellians may feel fewer moral concerns about not truthfully reporting own behavior [48].

### Narcissism

Individuals with pronounced *narcissistic* personality traits are self-absorbed and egocentric and have a constant need for validation for their inflated self-view [18]. Furthermore, narcissists believe in their superiority and tend to be dismissive of advice from others [54, 55].

With respect to the primary immunization, narcissistic individuals consider their own health as more important than the health of other individuals and may feel entitled to be prioritized to get vaccinated. They consider themselves as good leaders and role-models for others [56]. The initially scarce and new technology of vaccines can serve as a potential source of narcissistic supply and self-validation. Seeking affirmation satisfies the grandiose self-concept of narcissists [57]. At the same time, individuals with high narcissistic personality traits may show aggravated concern about potential side effects and may not want to serve as “guinea pigs” on a newly developed vaccination technology, which can reduce the likelihood of getting vaccinated. Even though narcissistic individuals are more self-centered, being vaccinated and potentially protecting others from harm may be a way to gain social recognition for protecting others. Overall, these described motives and the self-serving behavior may all be present and we therefore do not come up with a clear prediction whether narcissistic individuals are more or less likely to have received primary immunization.

With respect to the booster uptake, concerns about protecting others from infection were less pronounced in the marketing campaign. Due to the declining protection of the primary immunization over time and the requirement to booster, the COVID-19 vaccination may be considered disappointing by some individuals. We hypothesize that feeling deceived about the short-term nature of protection from COVID-19 is potentially more pronounced for individuals with high narcissistic personality traits. Narcissistic personality has been shown to be associated with low levels of forgiveness, see Exline et al. [58]. Accordingly, narcissistic individuals can be expected to be less forgiving – also with respect to a vaccination, which did not provide as long-lasting coverage as expected. In addition, booster vaccinations were not scarce anymore at the time of the study. A generally available vaccine offers less narcissistic supply. Overall, the booster vaccination had less appeal to narcissistic individuals than the primary immunizations and we therefore expect that they are more likely to opt out from booster vaccinations after having received a primary immunization.

With respect to simple preventive behavior, narcissistic individuals may receive self-validation from displaying extremely careful behavior towards others. They may feel more shame to admit that they do not always adhere to meaningful preventive behavior and may therefore over-report their preventive behavior. Accordingly, we expect narcissistic individuals to report more preventive behavior.

### Psychopathy

Pronounced *psychopathic* personality traits are associated with lack of empathy and remorse and bold, disinhibited behavior [18]. It is worth noting that psychopaths are more likely to act on temptation than Machiavellians and therefore show more impulsive behavior and more risk-taking [59]. Similarly, psychopathic individuals are also more pronounced to antisocial behavior and tend to aggression and physical threat [48, 59, 60].

Pro-social concerns of getting vaccinated and adhering to other simple preventive measures matter less to individuals with high psychopathic traits and their risk seeking tendencies can reduce the appeal of prevention. Accordingly, we predict that individuals with high psychopathic personality traits are less likely to get vaccinated, to receive a booster, and to engage in other preventive behavior.

In sum, as the three Dark Triad personality concepts show distinctive correlates we will investigate separate measurements in our analysis.

## Methods

### ECOS survey on COVID-19-related behavior

Our data utilizes wave nine from the European Covid Survey. This longitudinal study covers Germany, the United Kingdom, Denmark, Netherlands, France, Portugal, Italy, and Spain. In each country, data is collected from about 1,000 respondents. The initial wave of the web-based survey was conducted in April 2020. The respondents were recruited via the market research company Dynata. In order to ensure the representativeness with respect to gender, age, region, and education within each sample, country specific quotas based on the respective census data were used. We applied common quality controls, such as excluding individuals [61], who completed the questionnaire in less than a third of the median survey duration per country, as well as excluding multiple responses by the same person and incomplete responses. Respondents were paid by the panel provider for complete surveys, which is common practice in online data collections (see for example Smith et al. [13]). The questionnaire was programmed by the authors to avoid ordering effects by employing block randomizers and force response as a condition for the completion of the questionnaire to avoid missing data by design. A more detailed description of the ECOS survey and fieldwork can be found in Sabat et al. [62].

The data collection of wave nine started on 23 December 2021 and was completed on 10 January 2022. We restrict our analysis to Germany and the United Kingdom because the respective questionnaires include new questions on Dark Triad personality. Further details on the relevant parts of the questionnaire can be found in Appendix A.

**Fig. 1:**
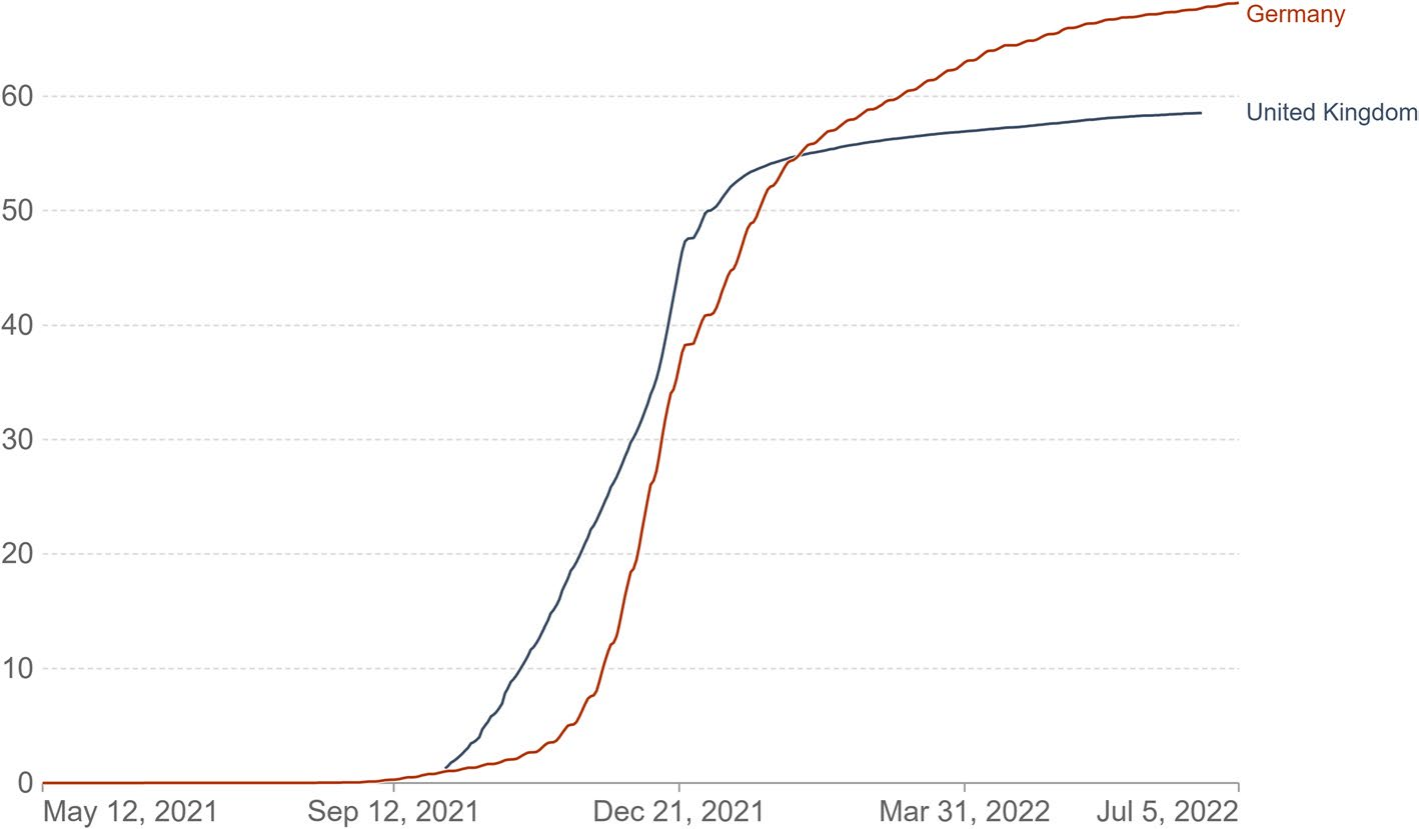
COVID-19 booster uptake in Germany and the UK. Total number of COVID-19 vaccine booster doses administered, divided by the total population of the country (in percentage). (Source: Official data collated by Our World in Data)

As our analysis is based on data from Germany and the UK, we focus on the timeline of COVID-19 in these two countries. Even though the coronavirus hit Germany and the UK just days apart at the end of January 2020, both countries responded differently (see for example Van Kessel et al. [63] for an overview of different COVID-19 vaccination policies in Europe). At the time the data for this study was collected, booster shots were already available and all adults were eligible. Nevertheless, the booster campaign in the UK was further advanced than in Germany and the booster rate in the UK was about 10 percentage points higher as depicted in Fig. 1.

### Measures

In our study, we investigate the association between dark personality traits and COVID-19-related preventive behavior, e.g. primary vaccination and booster uptake, and adherence to simple preventive measures. The respondent’s general COVID-19 vaccination acceptance (or hesitancy) is assessed with the actual vaccination status. The respondents indicate whether they have received no shot, the first shot or if they have already received primary immunization as they have received two shots or three shots (booster). The measure for simple preventive behavior comprises six items with four levels, covering the following aspects: regular hand washing; covering nose and mouth when sneezing; physical distancing; avoiding shaking hands, hugging, kissing; using alcohol-based hand rub; and avoiding touching the face. These measures reflect the initial recommendations by the WHO [28, 64] to reduce the spread of the pandemic and the individual risk of infection and became common measures of COVID-19 prevention in the literature (see for example Varghese et al. [44], Jordan, Yoeli, and Rand [65], Smith et al. [13], Wachira et al. [66]). Due to the panel nature of the ECOS data collection, the measures were repeatedly collected to allow comparisons over time. Similar to other COVID-19 related publications (see for example Smith et al. [13], Neumann-Böhme, Sabat, and Attema [45], Al-Zubaidy et al. [67]), we summarized the preventive behaviors in one index. The total adherence index was calculated by standardizing and adding up the scores of each item, divided by the number of items.

To explain COVID-19 vaccine-related decisions our variables of interest are malevolent personality traits, i.e. Machiavellianism, narcissism, and psychopathy. To assess Machiavellianism, narcissism, and psychopathy, the validated Short Dark Triad questionnaire (SD3) developed by Jones and Paulhus [49] is used. The Dark Triad score comprises nine items for each personality trait, totalling to 27 items. Answers can be given on a 5-point Likert-scale, ranging from 1 (strongly disagree) to 5 (strongly agree).^3^ The Dark Triad score was derived using principal component analysis analogous to the approach from Pailing, Boon, and Egan [72], which is widely used in the literature. This score combines the three Dark Triad personality traits into one scalar by multiplying each mean response per personality trait with the respective factor loading and add them up to one index. Throughout the analysis, we use this measure as the Dark Triad score. In addition, we investigate the three Dark Triad personalities separately. For each personality score, we use the mean of the standardized answers to nine items.

In addition, we control for socio-economic factors that have been found to be associated with COVID-19-related decisions and are therefore included into our model [6, 43, 73]. Socio-economic controls include *country of residence*, *age*, *gender*, relative *income* (making ends meet), *education*, *relationship status*, number of *children* in the household, and *risk groups* present in the household (e.g. elderly persons or someone with diagnosed chronic medical conditions). In addition, we include the *health risk attitude* by 11 categories. To capture the individual health risk attitude, participants have to rate their willingness to take health risks on an 11-point Likert-scale from 0 (not at all willing to take health risks) to 10 (very willing to take health risks). To measure the health state we use the *EQ-5D* (see Herdman et al. [74]) and calculate a score for the health status. Well-being is measured using the *ICECAP-A* (see Al-Janabi, Flynn, and Coast [75]), where we again standardize the answers to calculate a score for the quality of life.

### Analysis

We investigate the effect of the Dark Triad personality on vaccination uptake, e.g. to have received primary immunization and to already have received a booster shot (when having received the primary immunization) as well as adherence to simple preventive measures in response to COVID-19.

To estimate the effect of malevolent personality on vaccination uptake, our models are specified as follows:

1 $$\begin{aligned} Logit(Y_i) = \beta _0 + \beta _1 DarkTriad_i + \gamma X_{controls_i} + \epsilon _i\ \text {with}\ i=1,...,N \end{aligned}$$

2 $$\begin{aligned} Logit(Y_i) & = \beta _0 + \beta _1 psychopathy_i + \beta _2 narcissism_i + \beta _3 Machiavellianism_i \nonumber \\ & \quad + \gamma X_{controls_i} + \epsilon _i ~\text {with}~ i=1,...,N \end{aligned}$$

The dependent variable $Y_i$ is the vaccination status, i.e. having received primary immunization and having received a booster shot (when having received primary immunization). The set of control variables described above is denoted by $X_{controls_i}$. Socio-economic controls include country of residence, age, gender, income, education, the relationship status, a variable that indicates whether the household includes kids under the age of 18, a variable that indicates whether the household includes risk groups, self-reported health risk attitude, self-reported health status (EQ-5D), and self-reported quality of life (ICECAP-A). Our variable of interest in our first Logit estimation (1) is the Dark Triad score derived via principal component analysis.^4^ In addition, in model (2) we investigate the three Dark Triad personality scores, i.e. on Machiavellianism, narcissism, and psychopathy, separately.

In addition, we investigate the link between Dark Triad personality and simple preventive behavior like physical distancing or regular hand washing. We estimate the following random effects models:

3 $$\begin{aligned} Preventive\ behavior_{ij} = \beta _0 + \beta _1 DarkTriad_{ij} + \gamma X_{controls_{ij}} + \alpha _j + \epsilon _{ij} ~\text {with}~ i=1,...,N \end{aligned}$$

4 $$\begin{aligned} Preventive~behavior_{ij} & = \beta _0 + \beta _1 psychopathy_{ij} + \beta _2 narcissism_{ij} + \beta _3 Machiavellianism_{ij} \nonumber \\ & \quad + \gamma X_{controls_{ij}} + \alpha _j + \epsilon _{ij} ~\text {with}~ i=1,...,N \end{aligned}$$

The dependent variable is the preventive behavior score. To assess preventive behavior, we use the mean of the standardized responses to the six items on preventive behavior. Random effects on country level are included. Our set of control variables $X_{controls_{ij}}$ is the same as above. In addition, we include the vaccination status of the individual as an additional control. Again, in addition to investigating the Dark Triad score in model (3), we also account for each Dark Triad personality trait separately in model (4).

## Results

### Descriptive statistics

Our sample includes individual characteristics of 1,007 participants from Germany (DE) and 1,023 participants from the UK, totaling to 2,030 individuals. A small majority of the participants (52%) are female and 72% of the participants have no children. The mean age of the full sample is about 50 years. On average, 42% are able to make ends meet fairly easily, 44% have a high education level, and 49% are married.

In addition, our sample includes information on vaccination status, adherence to simple preventive measures, and Dark Triad personality. The majority of the sample have already received (at least) primary immunization as they have received two or three shots. On average, 86% (DE: 84%; UK: 87%) of the participants in our sample are primary vaccinated. In addition, most of the participants (whole sample: 61%; DE: 52%; UK: 70%) responded that they have already received a booster shot. Constraints on availability of and access to vaccination can be one explanation for the lower booster rate in Germany as the COVID-19 booster campaign was less evolved there compared to the UK. The mean (median) score of preventive behavior is 3.07 (3.17). With respect to the Dark Triad personality, individuals scored the highest on Machiavellianism. The overall Dark Triad score derived via principal component analysis ranges from −3.73 to 4.01. The Dark Triad personality scores do not differ substantially between Germany and the UK. Full tables on summary statistics are provided in Appendix B.

### Dark Triad personality and primary immunization

In the following sections, we report our regression results. In our first model specification the dependent variable $Y_i$ equals 1 if the individual has received primary immunization, as they have received two or three shots, and 0 if not. Therefore, our model estimates the likelihood of having received at least primary immunization. The control group are individuals who have received no shot or one shot. Table 2 shows estimation results from the logit model (1) and (2).

In column (1), the Dark Triad score is significant at the 5% level controlling for socio-economic factors. This implies that individuals scoring high at the SD3 are less likely primary immunized. An increase in the Dark Triad score of one unit is associated with a decrease in the likelihood of having received primary immunization of 1.29 percentage points. Our results in column (2) show that the negative correlation of SD3 and the likelihood of having received primary immunization is linked to psychopathy. The coefficient estimate is negative and significant at the 5% level, supporting the argument that individuals with higher psychopathic traits are less likely to get vaccinated. A one unit increase in the psychopathy score is associated with a decrease in the probability of having received primary immunization of 3.31 percentage points.

With regard to socio-economic controls, there is a positive relationship between age, income, the quality of life, and the likelihood of having received primary immunization. Individuals from the UK have significantly more likely received primary immunization which can be attributed to the earlier start of the vaccination campaign so that more people are already vaccinated at the timing of the survey. Being single and having kids has a significant negative correlation to vaccination acceptance. We report the full Table 2 including estimates on control variables in Appendix B.

**Table 2:** Dark Triad personality and primary immunization

	(1) Logit: Primary Vaccination	(2) Logit: Primary Vaccination
Dark Triad Score	−0.116** (0.056)	
*Marginal effects*	−1.29 pp**	
Psychopathy		−0.299** (0.146)
*Marginal effects*		−3.31 pp**
Narcissism		−0.077 (0.158)
*Marginal effects*		−0.86 pp
Machiavellianism		0.030 (0.129)
*Marginal effects*		0.34 pp
Additional controls	Gender, **Age**, **Country**, Education, **Income**, **Kids**, **Relationship Status**, Risk Group, Health Risk Attitude, EQ-5D, **ICECAP**	Gender, **Age**, **Country**, Education, **Income**, **Kids**, **Relationship Status**, Risk Group, Health Risk Attitude, EQ-5D, **ICECAP**
$N$	2,030	2,030
Pseudo $R^{2}$	0.103	0.105

Significance level indicate the following: * $p<0.10$, ** $p<0.05$, *** $p<0.01$. Standard errors are shown in parentheses and marginal effects (in percentage points) below. The table shows regression results using a logit model. The dependent variable is the vaccination status, e.g. having received primary immunization. The dependent variable is 1 if the individual has received primary immunization as having received two or three shots and 0 otherwise. In column (1) the *Dark Triad score* is calculated using principal component analysis, i.e. the mean answers to narcissism, psychopathy, and Machiavellianism are multiplied with the respective factor loading and added up to one scalar. In column (2) the score on *psychopathy* is calculated by averaging the (standardized) answers to nine questions. The scores on *narcissism* and *Machiavellianism* are calculated analogously

Because of the timing of wave nine of the survey (23 December 2021 to 10 January 2022), we have to account for the variation in eligibility of and access to vaccines in Germany and the UK. Compared to Germany (84%), we observe a higher vaccination rate with regard to having received primary immunization in the UK (87%) as the vaccination campaign was further evolved in this country. In addition, while the majority of people in Germany have a clear preference for the Biontech/Pfizer vaccine (58%), people tend to prefer the AstraZeneca vaccine in the UK (18%) compared to Germany (2%) in our sample.

As a robustness check, we investigate the effect of Dark Triad personality on having received primary immunization for each country, i.e. Germany and UK, separately. Table 3 provides estimation results on our first model specification for Germany in column (1) and (2) and the UK in column (3) and (4), where we compare individuals who have already received (at least) primary immunization to those who have not. While we find no significant relationship for Dark Triad personalities in Germany, in the UK the negative correlation between psychopathy and primary immunization is even more pronounced and significant at the 5% level, which can be attributed to the different development of the vaccination campaign. Interestingly, we observe a positive association for Machiavellian personality and having received primary immunization in the UK, but the effect is only marginally significant.

**Table 3:** Dark Triad personality and primary immunization by country

	(DE)		(UK)	
	(1) Logit: Prim Vacc	(2) Logit: Prim Vacc	(3) Logit: Prim Vacc	(4) Logit: Prim Vacc
Dark Triad Score	−0.112 (0.076)		−0.087 (0.088)	
*Marginal effects*	−1.39 pp		−0.81 pp	
Psychopathy		−0.163 (0.199)		−0.540** (0.226)
*Marginal effects*		−2.02 pp		−4.98 pp**
Narcissism		0.125 (0.210)		−0.182 (0.254)
*Marginal effects*		1.55 pp		−1.68 pp
Machiavellianism		−0.271 (0.179)		0.421** (0.194)
*Marginal effects*		−3.36 pp		3.88 pp**
Additional controls	Gender, **Age**, Education, **Income**, Kids, Relationship Status, Risk Group, Health Risk Attitude, EQ-5D **ICECAP**		Gender, **Age**, **Education**, **Income**, Kids, **Relationship Status**, **Risk Group**, Health Risk Attitude, EQ-5D ICECAP	
$N$	1,007	1,007	1,023	1,023
Pseudo $R^{2}$	0.071	0.073	0.183	0.194

Significance level indicate the following: * $p<0.10$, ** $p<0.05$, *** $p<0.01$. Standard errors are shown in parentheses and marginal effects (in percentage points) below. The table shows regression results using a logit model for Germany (column (1) and (2)) and the UK (column (3) and (4)) separately. The dependent variable is the vaccination status, e.g. having received primary immunization. The dependent variable is 1 if the individual has received primary immunization as having received two or three shots and 0 otherwise. In column (1) and (3) the *Dark Triad score* is calculated using principal component analysis, i.e. the mean answers to narcissism, psychopathy, and Machiavellianism are multiplied with the respective factor loading and added up to one scalar. In column (2) and (4) the score on *psychopathy* is calculated by averaging the (standardized) answers to nine questions. The scores on *narcissism* and *Machiavellianism* are calculated analogously

### Dark Triad personality and booster uptake

In a second model specification, the dependent variable $Y_i$ equals 1 if the individual has already received a booster shot and 0 if not. The control group in this setting are individuals who have already received the primary vaccination. Therefore, our model estimates the likelihood of having received a booster shot when already having received primary immunization. Table 4 reports the regression results from logit model (1) and (2).

With respect to having already received a booster shot, the coefficient on Dark Triad is negative as well but not significant. In column (2), narcissism is significant at the 5% level, while psychopathy and Machiavellianism are not significant. Narcissistic individuals are associated with a higher likelihood of refusing booster shots after having received the primary vaccination. Specifically, a one unit increase in the score on narcissism is associated with a decrease in the probability of having received a booster shot (after having received primary immunization) of 4.52 percentage points. Neither do we find a significant correlation for Machiavellianism on the probability of already having received a booster shot nor for psychopathy.

Among utilized socio-economic controls, there is a positive relationship between age, education, income as well as the effect of risk groups in the household and the probability of being boostered (after having received primary immunization). Again, individuals from the UK are more likely boostered than individuals from Germany. Being single and having kids is significantly negatively correlated with booster uptake. We report the full Table 4 including estimates on control variables in Appendix B.

**Table 4:** Dark Triad personality and booster uptake

	(1) Logit: Booster	(2) Logit: Booster
Dark Triad Score	−0.042 (0.050)	
*Marginal effects*	−0.69 pp	
Psychopathy		0.142 (0.128)
*Marginal effects*		2.32 pp
Narcissism		−0.276** (0.138)
*Marginal effects*		−4.52 pp**
Machiavellianism		−0.028 (0.114)
*Marginal effects*		−0.46 pp
Additional controls	Gender, **Age**, **Country**, **Education**, **Income**, **Kids**, **Relationship Status**, **Risk Group**, Health Risk Attitude, EQ-5D, ICECAP	Gender, **Age**, **Country**, **Education**, **Income**, **Kids**, **Relationship Status**, **Risk Group**, Health Risk Attitude, EQ-5D, ICECAP
$N$	1,740	1,740
Pseudo $R^{2}$	0.174	0.176

Significance level indicate the following: * $p<0.10$, ** $p<0.05$, *** $p<0.01$. Standard errors are shown in parentheses and marginal effects (in percentage points) below. The table shows regression results using a logit model. The dependent variable is the vaccination status, e.g. having received a booster shot (when having received primary immunization). The dependent variable is 1 if the individual has received a booster shot and 0 if the individual has received primary immunization but no booster shot. In column (1) the *Dark Triad score* is calculated using principal component analysis, i.e. the mean answers to narcissism, psychopathy, and Machiavellianism are multiplied with the respective factor loading and added up to one scalar. In column (2) the score on *psychopathy* is calculated by averaging the (standardized) answers to nine questions. The scores on *narcissism* and *Machiavellianism* are calculated analogously

Again, because of the timing of wave nine of the survey, we have to account for the variation in eligibility of and access to booster in Germany and the UK. Compared to Germany, we observe a higher booster rate in the UK as the booster campaign was further evolved in this country. As a robustness check, we investigate the effect of Dark Triad personality on booster uptake for Germany and the UK, separately. Table 5 provides estimation results on our second model specification for Germany in column (1) and (2) and the UK in column (3) and (4). With regard to the relationship between Dark Triad personality and booster uptake in Germany, again we do not find any significant association controlling for socio-economic factors. If we only consider individuals from the UK in column (3) and (4), we find significant and even more pronounced effects of the Dark Triad personality on booster uptake compared to the results based on the full sample. Specifically, a one unit increase in the Dark Triad score is associated with a decrease in the probability of having received a booster shot (after having received primary immunization) of 1.95 percentage points. With regard to the three Dark Triad personality traits, again, we find a significant association for narcissism at the 5% level. A one unit increase in the score on narcissism is associated with a decrease in the probability of having received a booster shot of 5.92 percentage points. One reason for the more pronounced effect of narcissism on booster hesitancy in the UK can be due to the fact that more people preferred the AstraZeneca vaccine (18%) compared to Germany (2%). After the arising debate on the effectiveness of the AstraZeneca vaccine, people with narcissistic personality may even be more disappointed after having received primary immunization and are therefore more likely to opt out from the booster shot.

**Table 5:** Dark Triad personality and booster uptake by country

	(DE)		(UK)	
	(1) Logit: Booster	(2) Logit: Booster	(3) Logit: Booster	(4) Logit: Booster
Dark Triad Score	0.038 (0.064)		−0.166* (0.086)	
*Marginal effects*	0.76 pp		−1.95 pp*	
Psychopathy		0.193 (0.171)		−0.065 (0.207)
*Marginal effects*		3.88 pp		−0.76 pp
Narcissism		−0.023 (0.180)		−0.506** (0.239)
*Marginal effects*		−0.47 pp		−5.92 pp**
Machiavellianism		−0.067 (0.152)		0.037 (0.184)
*Marginal effects*		−1.35 pp		0.43 pp
Additional controls	Gender, **Age**, **Education**, Income, **Kids**, **Relationship Status**, Risk Group, Health Risk Attitude, **EQ-5D** **ICECAP**		Gender, **Age**, Education, **Income**, Kids, **Relationship Status**, Risk Group, Health Risk Attitude, EQ-5D ICECAP	
$N$	847	847	893	893
Pseudo $R^{2}$	0.114	0.115	0.264	0.267

Significance level indicate the following: * $p<0.10$, ** $p<0.05$, *** $p<0.01$. Standard errors are shown in parentheses and marginal effects (in percentage points) below. The table shows regression results using a logit model for Germany (column (1) and (2)) and the UK (column (3) and (4)) separately. The dependent variable is the vaccination status, e.g. having received a booster shot (when having received primary immunization). The dependent variable is 1 if the individual has received a booster shot and 0 if the individual has received primary immunization but no booster shot. In column (1) and (3) the *Dark Triad score* is calculated using principal component analysis, i.e. the mean answers to narcissism, psychopathy, and Machiavellianism are multiplied with the respective factor loading and added up to one scalar. In column (2) and (4) the score on *psychopathy* is calculated by averaging the (standardized) answers to nine questions. The scores on *narcissism* and *Machiavellianism* are calculated analogously

### Dark Triad personality and simple preventive behavior

This section shows regression results where we investigate the link between Dark Triad personality and simple preventive behavior like physical distancing or regular hand washing. Table 6 reports regression results using a random effects model investigating the Dark Triad score in column (1) and each Dark Triad personality trait separately in column (2). The dependent variable is the preventive behavior score.

The coefficient estimates for psychopathy and narcissism are significant at the 1% level. Specifically, individuals scoring high at psychopathy are associated with significantly lower adherence to simple preventive behavior. In contrast to this finding, individuals with higher narcissistic personality are more likely to adhere to preventive behavior practices. Specifically, a one unit increase in the score of psychopathy is associated with a decrease in the score on preventive behavior of 0.211 points, while a one unit increase in the score on narcissism is associated with an increase in the adherence-score on preventive behavior of 0.115 points. This result seems to be robust throughout the six preventive recommendations, even if considerable heterogeneity can be observed with regard to adherence to each measure [44] (not shown in the table). With respect to socio-economic factors, being older, a higher reported health status and quality of life significantly correlates with an increase in adherence to preventive measures, while being male, a higher income, being single, and a higher self-reported willingness to take health risks is associated with lower adherence. The association for health status is only significant in column (1). Having received a booster shot (compared to having received primary immunization) is significantly associated with higher adherence to simple preventive measures, while not having received primary immunization (compared to having received primary immunization) correlates negatively with engagement into other preventive behavior measures. Again, we report the full Table 6 including estimates on control variables in Appendix B.

**Table 6:** Dark Triad personality and preventive behavior

	(1) RE: Preventive Behavior	(2) RE: Preventive Behavior
Dark Triad Score	0.009 (0.039)	
Psychopathy		−0.211*** (0.008)
Narcissism		0.115*** (0.015)
Machiavellianism		0.128 (0.100)
Additional controls	**Gender**, **Age**, **Vaccination Status**, Education, **Income**, Kids, **Relationship Status**, Risk Group, **Health Risk Attitude**, **EQ-5D** **ICECAP**	**Gender**, **Age**, **Vaccination Status**, Education, **Income**, Kids, **Relationship Status**, Risk Group, **Health Risk Attitude**, EQ-5D **ICECAP**
$N$	2,030	2,030

Heteroskedasticity-robust standard errors in parentheses, * $p<0.10$, ** $p<0.05$, *** $p<0.01$. Note: The table shows regression results using a random effects model with region random effects on country level. The dependent variable is a score on adherence to preventive behavior. Adherence to preventive behavior is assessed by averaging the (standardized) answers to six questions covering the following aspects: regular hand washing, covering nose and mouth when sneezing, physical distancing, avoiding shaking hands/hugging/kissing, using alcohol-based hand rub, and avoiding touching the face. In column (1) the *Dark Triad score* is calculated using principal component analysis, i.e. the mean answers to narcissism, psychopathy, and Machiavellianism are multiplied with the respective factor loading and added up to one scalar. In column (2) the score on *psychopathy* is calculated by averaging the (standardized) answers to nine questions. The scores on *narcissism* and *Machiavellianism* are calculated analogously

To summarize, we find evidence that Dark Triad personality traits are significantly associated with primary vaccination and booster uptake as well as adherence to simple preventive measures. Therefore, our results highlight an important channel of vaccination hesitancy in addition to factors that have already been found to be associated with COVID-19-related behavior in previous studies. In addition, our results suggest to account for distinct aspects of the Dark Triad personality as psychopathy, narcissism, and Machiavellianism show different patterns of correlation with vaccination uptake and socially responsible behavior.

In addition, we conduct further sensitivity analyses in which we account for potential multicollinearity between the Dark Triad personality traits, restrict our sample, rerun the regression estimating a probit model, and use an alternative score as the Dark Triad score to ensure the robustness of our results presented in this chapter. Our findings show extremely robust estimates. Sensitivity analyses are shown in Appendix C.

## Discussion

This paper investigated the relationship between the Dark Triad personality traits, vaccinations against COVID-19, and adherence to (simple) preventive behaviors. We find that dark personality traits correlate with the demand for vaccines, indicated by the vaccination status for both the primary and the booster vaccination as well as the adherence to other simple preventive measures in different ways. These findings suggest that tailored communication and delivery strategies may be beneficial in addressing potential psychological barriers to vaccination uptake.

The global COVID-19 pandemic has not only been a stress test for hospitals, outpatient services, and health administrations, but has also changed societies (see Vicente and Suleman [76] for an overview). For example, results from the US suggest that the pandemic highlighted the selfless behavior of others and a mutual dependence in society [77]. At the beginning of the pandemic, the public goods perspective of prevention was heavily emphasized: Individuals were asked to stay at home to protect elderly from harm, and vaccinations were also marketed as not only protecting the vaccinated person but also stopping the spread of the pandemic. Personality traits heavily influence our willingness to do things that benefit others. The literature on personality traits and COVID-19 behaviors suggests that individual risk perception and cognitive biases [78], personality differences [79], and individualism [80] play a role in adherence to social distancing and containment measures. Psychological characteristics such as conspiratorial and paranoid beliefs were related to vaccination hesitancy [20] and altruism plays a role in pro-social pandemic behaviors [45].

In this study, we find evidence that Dark Triad personality traits are associated with preventive behaviors with respect to the COVID-19 pandemic in several ways. These findings relate to earlier studies that for example link the DT personality traits, COVID-19-specific conspiracy beliefs and the willingness to be vaccinated in the future [24]. In the following, we discuss our results based on the predictions for each personality trait outlined in the Motivation section.

We expected that individuals with high scores in Machiavellian personality traits will be more likely to adhere to preventive measures and not differ from the rest of the sample regarding primary and booster vaccinations. As expected, we did not find a relationship between Machiavellianism and vaccination hesitancy but, interestingly, Konc, Petrović, and Dinić [81] showed that deviousness (an aspect of Machiavellianism) was associated with an unwillingness to vaccinate against COVID-19 independently of risk-taking tendencies. In contrast to the a priori expectations, we did not find a significant association between Machiavellianism and adherence to simple preventive behaviors.

Our prediction for narcissism was that respondents with narcissistic personality traits will not differ from the rest of the sample in terms of primary immunizations, but are more likely to opt out of booster shots in part because these were not scarce anymore and provide less narcissistic supply. We further predicted that respondents with higher narcissism scores were more likely to report adherence to preventive measures because they may receive self-validation from displaying extremely careful behavior towards others. As expected, we find no significant relationship between narcissism and primary vaccinations. In line with expectations, our findings show that narcissistic personality traits are associated with a higher likelihood of refusing booster shots after receiving the primary vaccinations. The differences are economically relevant, with marginal effects of 4.52 percentage points. In line with our findings, narcissism and psychopathy appear to be related to vaccination hesitancy and anti-vaccine outcomes [82]. Interestingly, Li and Cao [83] extend the dark personality traits and find that (everyday) sadism was also associated with more vaccine refusal. In line with our predictions, we find that higher scores of narcissism are positively associated with preventive behaviors.

Since psychopathy is associated with a lack of empathy and remorse as well as bold, disinhibited behavior, we expected respondents with high psychopathy scores to be unwilling to be vaccinated as well as less likely to engage in simple preventive behaviors. Contrary to our expectations, we did not find a significant relationship between psychopathy and booster vaccinations, while our other two predictions on psychopathy can be supported by the ECOS data. In line with the predictions, we find that individuals who scored higher in psychopathy were significantly less likely to have received primary immunization with marginal effects of 3.31 percentage points. One reason why we did not find a significant relationship for booster hesitancy is that psychopathic individuals may have already dropped out for primary immunization, as we only consider individuals that have at least received primary immunization to analyze booster uptake. Furthermore, individuals with higher psychopathic tendencies are associated with a lower engagement in simple preventive behaviors, which supports Konc, Petrović, and Dinić [81] findings, who show the same effect for recklessness as an aspect of psychopathy.

Overall, our results are almost completely in line with the theoretical predictions we made a priori about the three personality types and relate to earlier findings in the literature.

This study adds to the growing understanding of vaccination hesitancy and adherence to COVID-19 preventive measures. Our findings highlight the crucial role that personality traits, specifically the Dark Triad, play in these behaviors. The importance of addressing certain personality traits in (public) information campaigns to increase the effectiveness of the advertisement is well established in the marketing literature, for example in the domain of road safety advertising [84] or in the context of tailoring political advertisements towards voter’s personality traits [85]. In a health setting, Olsacher et al. [86] find a correlation between the big 5 personality traits and organ donation, with a higher effectiveness of messages that were tailored towards the personality traits. Related to COVID-19 and the dark triad personality traits, Huang, Yang, and Dai [87] find that people who score higher in Machiavellianism reported stronger anti-pandemic intentions (e.g. wanting to learn more about COVID-19) when a message was tailored towards the impact of COVID-19 on themselves compared to messages highlighting the impact towards others. Policymakers, health professionals, and those in charge of public health messaging may take these factors into account when devising communication strategies to improve the vaccination uptake and adherence to preventive behaviors.

Firstly, to address narcissistic tendencies, messaging around booster vaccines should be tailored to appeal to individuals’ sense of uniqueness and importance. Strategies could include emphasizing the advanced technology used in the booster shots, making it a ’premium’ choice. This approach could also leverage on exclusivity or status to appeal to the inherent desire for admiration that characterizes this personality trait.

Secondly, our findings show that individuals with psychopathic tendencies are associated with a lower likelihood of getting vaccinated and adhering to preventive measures. Given the spontaneous and thrill-seeking nature associated with high psychopathy levels, alternative approaches could be explored to target this group. One could consider spontaneous vaccination offers in environments that appeal to their impulsivity, such as casinos or theme parks. Quick-access, walk-in vaccination centers, or pop-up clinics in highly trafficked areas might also provide the spontaneity that would appeal to such individuals.

Lastly, there is a need for greater public education and transparent communication to tackle the influence of conspiracy theories, which is particularly relevant for individuals with high dark triad personality traits due to the greater susceptibility to conspiracy theories. Policymakers should prioritize evidence-based, accessible information to debunk common myths and conspiracies surrounding COVID-19 vaccinations.

Some limitations need emphasizing: First, we used self-reported vaccination status from a representative online survey and are not able to verify the official status. Respondents with high scores in the Dark Triad personality traits could be more likely to misrepresent their answers. At the same time, the anonymity and online nature of the study provided no incentives to provide socially desirable answers and we find a substantial difference in vaccination decisions based on the Dark Triad personality traits psychopathy and narcissism. Furthermore, employing online panels to elicit the populations preferences was a common and useful way to attain data in the pandemic. Nonetheless, it came with certain drawbacks, for example problems to reach older parts of the population and the risk of people filling out these surveys professionally. We carefully designed the online questionnaire to avoid that respondents were unable to complete the questionnaire, i.e. because of a lack of understanding of questions or, on the other hand, that some respondents speed through the survey to be rewarded without providing meaningful information. We therefore had to make certain design decisions such as the use of the force response option to avoid missing data. Since we provided middle options such as “neither agree nor disagree”, we believe respondents had options to express their preferences to continue. Second, as indicated in figure 1, the vaccination campaigns in the UK and Germany differed concerning the availability of vaccines due to the earlier Conditional Marketing Authorization of vaccines in the UK, which may have resulted in higher reported vaccination rates in the UK in this study. We find a higher association of Dark Triad personality traits with booster vaccinations, which can be due to the differences in roll-out. Lastly, we caution the reader to interpret our results as correlations rather than causal effects as the observed behavior itself may influence or even reinforce personality traits. While existing literature predominantly suggests Dark Triad personalities to be rather stable or slightly decreasing over longer time horizons (see for example Edelstein, Newton, and Stewart [88], Lynam et al. [89], Grosz et al. [90]), we cannot fully rule out that COVID-19 preventive measures may influence self-reporting of personality traits. While our findings indicate statistically significant associations, we acknowledge that other unobserved factors may contribute to these relationships. Future longitudinal or experimental studies could provide deeper insights into the directional effects of personality traits on health-related decision-making.

## Conclusions

Our results suggest that personality types should be considered for such information campaigns to frame messages and adopt policies that appeal for example to narcissists, because even small changes in messaging have shown to affect the level of adherence of different personality types [91]. These findings provide insights for future outbreaks and pandemics to achieve higher vaccination rates and more adherence to protective measures. Lastly, the relevance of psychopathic personality traits and the association with vaccine related conspiracy theories [24] may explain some of the radical intensity in the public and private debates about the COVID-19 vaccination.

In conclusion, there is a necessity of a targeted, nuanced approach to public health messaging to appeal to parts of the population that score high in the Dark Triad personality traits. While broad, universal messaging serves a purpose, acknowledging the impact of personality traits on health behaviors has the potential to reach and convince groups who might otherwise stay unwilling to be vaccinated. Future pandemics and public health crises would benefit from this understanding to promote greater public adherence to protective measures and lower levels of vaccination hesitancy.

## Supplementary Information

Supplementary Material 1: Appendix A.

Supplementary Material 2: Appendix B.

Supplementary Material 3: Appendix C.

## Data Availability

No datasets were generated or analysed during the current study.

## Footnotes

1 We observe intercorrelations of about 40% to 55% in our data. The highest correlation between narcissism and psychopathy is consistent with the original study from Paulhus and Williams [18].

2 The term pandemic fatigue is defined by WHO as the unwillingness to comply with recommended protective behavior for expanded time periods.

3 The SD3 questionnaire has been translated into German for use with German-speaking individuals. The German version of the SD3 serves as a reliable proxy for German samples, demonstrating high cross-language replicability, as evidenced by several studies (see for example Malesza et al. [68], Wehner et al. [69]) and was also assessed in other (cross-cultural) studies [70, 71].

4 However, our results are the same if we use the mean of all personality responses for each individual as the Dark Triad score instead.
