# Supplementary material for: Impact of Dark Triad personality traits on COVID-19 vaccination uptake and prevention efforts: insights from the European Covid Survey (ECOS)
Source: BMC Public Health. 2025 Apr 10;25:1352. doi: 10.1186/s12889-025-22471-3 (PMC11984049; doi:10.1186/s12889-025-22471-3)
Supplement: Supplementary file 1 — Supplementary Material 1: Appendix A. [file 12889_2025_22471_MOESM1_ESM.pdf]

Appendix A

COVID-19 vaccination acceptance

Have you already received a vaccination against COVID-19?

- Yes, the first shot
- Yes, with two shots
- Yes, with three shots (booster)
- Not yet, but I intend to
- No

Short Dark Triad (SD3)

Please indicate for each of the questions below, how much you agree / disagree with the statement. (on scale from 1 (disagree strongly), 2 (disagree), 3 (neither agree nor disagree), 4 (agree) to 5 (agree strongly))

Machiavellianism

1. It's not wise to tell your secrets.
2. I like to use clever manipulation to get my way.
3. Whatever it takes, you must get the important people on your side.
4. Avoid direct conflict with others because they may be useful in the future.
5. It's wise to keep track of information that you can use against people later.
6. You should wait for the right time to get back at people.
7. There are things you should hide from other people to preserve your reputation.
8. Make sure your plans benefit yourself, not others.
9. Most people can be manipulated.

Narcissism

1. People see me as a natural leader.
2. I hate being the center of attention.
3. Many group activities tend to be dull without me.
4. I know that I am special because everyone keeps telling me so.
5. I like to get acquainted with important people.
6. I feel embarrassed if someone compliments me.
7. I have been compared to famous people.
8. I am an average person.
9. I insist on getting the respect I deserve.

Psychopathy

1. I like to get revenge on authorities.
2. I avoid dangerous situations.
3. Payback needs to be quick and nasty.
4. People often say I'm out of control.
5. It's true that I can be mean to others.
6. People who mess with me always regret it.
7. I have never gotten into trouble with the law.
8. I enjoy having sex with people I hardly know.
9. I'll say anything to get what I want.

Simple Preventive Behavior

Next, we would like to know about your own practices related to the novel coronavirus. Thinking about the last eight weeks, did you adhere to the following activities due to concerns about the novel coronavirus? (on a scale from 1 (No), 3 (Yes, a bit), 4 (Yes, quite strongly) to 5 (Yes, fully))

- Regularly wash my hands with soap for at least 20 seconds.
- Cover my nose and mouth when coughing or sneezing.
- Keep a distance of at least 1 meter from other people.
- Avoid shaking hands, hugging or kissing when greeting others.
- Use alcohol-based hand rub.
- Avoid touching my nose, eyes and mouth.

Risk Group in Household

Does your household include any of the following members (other than you)?

- Very young children and babies
- Children
- **Disabled person(s)**
- **Someone with diagnosed chronic medical conditions (such as heart or lung conditions or diabetes)**
- **Elderly person(s)**
- None of the ones mentioned above

Health Risk Attitude

Are you generally a person who is willing to take health risks or do you try to avoid taking health risks? (from 0 = "not at all willing to take health risks" to 10 = "very willing to take health risks")

Health Status (EQ5D)

Now, we would like you to rate your own health.
(no problems, slight problems, moderate problems, severe problems, unable to do something)
Under each heading, please click the ONE box that best describes your health TODAY.

- Mobility
- Self-care
- Usual activities (e.g. work, study, housework, family or leisure activities)
- Pain / discomfort
- Anxiety / depression

Quality of Life (ICECAP)

We would like to know about your overall quality of life.
(a lot/completely, quite a lot/many, a little/a few, not any/unable)
Please indicate which statements best describe your overall quality of life at the moment.

- Feeling settled and secure
- Love, friendship and support
- Being independent
- Achievement and progress
- Enjoyment and pleasure
