# Supplementary material for: Impact of Dark Triad personality traits on COVID-19 vaccination uptake and prevention efforts: insights from the European Covid Survey (ECOS)
Source: BMC Public Health. 2025 Apr 10;25:1352. doi: 10.1186/s12889-025-22471-3 (PMC11984049; doi:10.1186/s12889-025-22471-3)
Supplement: Supplementary file 2 — Supplementary Material 2: Appendix B. [file 12889_2025_22471_MOESM2_ESM.pdf]

Appendix B

Table 7: Summary statistics: demographics

	Mean	Median	Min	Max	SD
DE	1,007				
UK	1,023				
Age	49.46	50.00	18.00	96.00	16.33
18-24	0.07	0.00	0.00	1.00	0.26
25-34	0.15	0.00	0.00	1.00	0.36
35-44	0.18	0.00	0.00	1.00	0.38
45-54	0.19	0.00	0.00	1.00	0.39
55-64	0.17	0.00	0.00	1.00	0.37
65+	0.24	0.00	0.00	1.00	0.43
Male	0.48	0.00	0.00	1.00	0.50
Income (great diff)	0.07	0.00	0.00	1.00	0.25
Income (some diff)	0.33	0.00	0.00	1.00	0.47
Income (fairly easily)	0.42	0.00	0.00	1.00	0.49
Income (easily)	0.18	0.00	0.00	1.00	0.39
Low education	0.16	0.00	0.00	1.00	0.37
Mid education	0.40	0.00	0.00	1.00	0.49
High education	0.44	0.00	0.00	1.00	0.50
Single	0.27	0.00	0.00	1.00	0.44
Living apart	0.04	0.00	0.00	1.00	0.21
Couple	0.14	0.00	0.00	1.00	0.34
Married	0.49	0.00	0.00	1.00	0.50
Widowed	0.03	0.00	0.00	1.00	0.18
Other civil status	0.03	0.00	0.00	1.00	0.17
No kids	0.72	1.00	0.00	1.00	0.45
One child	0.15	0.00	0.00	1.00	0.36
Two children	0.09	0.00	0.00	1.00	0.29
Three or more children	0.03	0.00	0.00	1.00	0.18
Risk group	0.28	0.00	0.00	1.00	0.45
Health risk attitude	3.51	3.00	0.00	10.00	2.76
Health status (EQ-5D)	4.41	4.60	1.00	5.00	0.72
Quality of life (ICECAP-A)	2.98	3.00	1.00	4.00	0.63
Observations	2,030				

Note: The table provides a descriptive summary (mean, median, minimum, maximum, and standard deviation) of socio-economic factors. Socio-economic factors include *country of residence* (Germany or United Kindom), *age*, dummy variables for *age categories*, a dummy variable *male* that is 1 for male individuals, dummy variables for *income categories* (defined as the household is able to make ends meet with great difficulty, with some difficulty, fairly easily, and easily), dummy variables for *education levels*, dummy variables for the *relationship status*, a dummy variable for whether the household includes *kids* under the age of 18, and a dummy variable *risk group* that is 1 if the household includes vulnerable individuals or individuals at risk (e.g. elderly persons or someone with diagnosed chronic medical conditions). To capture the individual *health risk attitude* participants have to rate their willingness to take health risks on an 11-point Likert-scale. *EQ-5D* assesses the self-reported health status in terms of "mobility", "self-care", "usual activities" (e.g. work, study or leisure activities), "pain/discomfort", and "anxiety/depression" on a 5-point Likert-scale. *ICECAP-A* assesses the self-reported quality of life with regard to "feeling settled and secure", "love, friendship and support", "being independent", "achievement and progress", and "enjoyment and pleasure" on a 4-point Likert-scale.

Table 8: Summary statistics: vaccination status, preventive behavior, and Dark Triad personality

	Mean	Median	Min	Max	SD
Primary immunization	0.86	1.00	0.00	1.00	0.35
Booster	0.61	1.00	0.00	1.00	0.49
Simple preventive measures	3.07	3.17	1.00	4.00	0.74
Dark Triad Score	0.00	0.04	-3.73	4.01	1.41
Narcissism	-0.00	0.09	-1.55	1.61	0.55
Machiavellianism	-0.00	-0.03	-1.93	1.79	0.62
Psychopathy	-0.00	-0.04	-1.09	1.68	0.67
Observations	2,030				

Note: The table provides a descriptive summary (mean, median, minimum, maximum, and standard deviation) of the vaccination status, adherence to simple preventive measures, and Dark Triad personality in our sample. The dummy variable *primary immunization* indicates whether an individual is fully vaccinated as having received two or three shots. The dummy variable *booster* indicates whether the individual has already received a booster shot. Adherence to *simple preventive measures* is assessed by averaging the (unstandardized) answers to six questions covering the following aspects: regular hand washing, covering nose and mouth when sneezing, physical distancing, avoiding shaking hands/hugging/kissing, using alcohol-based hand rub, and avoiding touching the face. The *Dark Triad score* was derived using principal component analysis and covers 27 items while *narcissism, Machiavellianism*, and *psychopathy* are the average scores of standardized answers to nine questions per personality trait.

Table 9: Dark Triad personality and primary immunization (full table 2)

	(1)<br>Logit: Primary Vaccination	(2)<br>Logit: Primary Vaccination
Dark Triad Score	-0.116** (0.056)	
Psychopathy		-0.299** (0.146)
Narcissism		-0.077 (0.158)

Machiavellianism		0.030 (0.129)
Health risk 1	0.341 (0.276)	0.341 (0.277)
Health risk 2	0.176 (0.257)	0.189 (0.258)
Health risk 3	0.218 (0.276)	0.262 (0.278)
Health risk 4	0.105 (0.308)	0.155 (0.310)
Health risk 5	0.136 (0.242)	0.205 (0.246)
Health risk 6	0.923*** (0.348)	0.996*** (0.351)
Health risk 7	0.231 (0.292)	0.292 (0.294)
Health risk 8	0.218 (0.306)	0.281 (0.308)
Health risk 9	-1.042** (0.412)	-0.994** (0.414)
Health risk 10	-0.549 (0.453)	-0.477 (0.456)
Male	-0.010 (0.141)	0.019 (0.142)
UK	0.319** (0.143)	0.324** (0.145)
25-34	0.395* (0.236)	0.392 (0.237)*
35-44	0.652*** (0.241)	0.638*** (0.241)
45-54	1.088*** (0.250)	1.055*** (0.251)
55-64	1.492*** (0.284)	1.456*** (0.285)
65+	1.429*** (0.280)	1.373*** (0.283)
Mid education	0.083 (0.195)	0.079 (0.196)
High education	0.273 (0.205)	0.264 (0.206)
Income (some diff)	0.462** (0.231)	0.461** (0.231)
Income (fairly easily)	0.881*** (0.241)	0.881*** (0.241)
Income (easily)	1.073*** (0.292)	1.076*** (0.292)
Single	-0.352** (0.153)	-0.347** (0.153)
Kids	-0.292* (0.166)	-0.279* (0.166)
Risk group	0.179 (0.169)	0.168 (0.169)
EQ-5D	-0.102 (0.091)	-0.122 (0.092)
ICECAP-A	0.185* (0.098)	0.184* (0.100)
cons	-0.020 (0.359)	-0.036 (0.360)
N	2,030	2,030
pseudo R^2	0.103	0.105

Standard errors in parentheses, * $p < 0.10$, ** $p < 0.05$, *** $p < 0.01$.

Note: The table shows regression results using a logit model. The dependent variable is the vaccination status, e.g. having received primary immunization. The dependent variable is 1 if the individual has received primary immunization as having received two or three shots and 0 otherwise. In column (1) the *Dark Triad score* is calculated using principal component analysis, i.e. the mean answers to narcissism, psychopathy, and Machiavellianism are multiplied with the respective factor loading and added up to one scalar. In column (2) the score on *psychopathy* is calculated by averaging the (standardized) answers to nine questions. The scores on *narcissism* and *Machiavellianism* are calculated analogously. *A*dditional controls include a dummy variable for the *gender* that is 1 for male individuals, dummy variables for *age* categories (with the age category 18-24 as the reference group), a dummy variable for the *country* (UK or Germany), dummy variables for *education* levels (with low education as a reference group), dummy variables for *income* (with being able to make ends meet with great difficulty as a reference group), a dummy variable for the *relationship status* that is 1 if the individual is single and lives alone and 0 if not, a dummy variable that indicates if the household includes *kids* under the age of 18, a dummy variable *risk group* that is 1 if the household includes individuals who belong to a risk group, i.e. elderly person(s) or someone with diagnosed chronic medical conditions such as heart or lung conditions or diabetes, self-reported *health risk attitude*, the variable *EQ-5D* that comprises the mean of (standardized) answers to five health related questions, and the variable *ICECAP* that comprises the mean of (standardized) answers to five questions on quality of life.

Table 10: Dark Triad personality and booster uptake (full table 4)

	(1)<br>Logit: Booster	(2)<br>Logit: Booster
Dark Triad Score	-0.042 (0.050)	
Psychopathy		0.142 (0.128)
Narcissism		-0.276** (0.138)
Machiavellianism		-0.028 (0.114)
Health risk 1	0.164 (0.232)	0.151 (0.233)
Health risk 2	0.112 (0.229)	0.104 (0.229)
Health risk 3	0.333 (0.244)	0.315 (0.246)
Health risk 4	-0.039 (0.279)	-0.061 (0.281)
Health risk 5	-0.136 (0.218)	-0.172 (0.221)
Health risk 6	-0.157 (0.256)	-0.189 (0.259)
Health risk 7	-0.037 (0.266)	-0.065 (0.268)
Health risk 8	-0.245 (0.283)	-0.293 (0.285)
Health risk 9	0.037 (0.521)	-0.020 (0.521)
Health risk 10	0.247 (0.525)	0.181 (0.525)
Male	0.112 (0.127)	0.094 (0.127)
UK	1.013*** (0.128)	0.969*** (0.131)
25-34	0.058 (0.253)	0.056 (0.253)
35-44	0.665*** (0.253)	0.670*** (0.253)
45-54	1.289*** (0.252)	1.299*** (0.253)
55-64	1.621*** (0.265)	1.647*** (0.266)
65+	2.044*** (0.275)	2.080*** (0.277)
Mid education	0.467*** (0.176)	0.461*** (0.177)
High education	0.588*** (0.183)	0.611*** (0.184)

Income (some diff)	0.608** (0.252)	0.608** (0.253)
Income (fairly easily)	0.553** (0.254)	0.551** (0.254)
Income (easily)	1.102*** (0.291)	1.109*** (0.292)
Single	-0.371*** (0.140)	-0.378*** (0.140)
Kids	-0.398*** (0.153)	-0.391** (0.153)
Risk group	0.303** (0.149)	0.305** (0.149)
EQ-5D	-0.107 (0.084)	-0.095 (0.085)
ICECAP-A	0.073 (0.088)	0.102 (0.090)
cons	-1.639*** (0.377)	-1.607*** (0.377)
N	1,740	1,740
pseudo R^2	0.174	0.176

Standard errors in parentheses, * $p < 0.10$, ** $p < 0.05$, *** $p < 0.01$.
Note: The table shows regression results using a logit model. The dependent variable is the vaccination status, e.g. having received a booster shot (when having received primary immunization). The dependent variable is 1 if the individual has received a booster shot and 0 if the individuals has received primary immunization but no booster shot. In column (1) the *Dark Triad score* is calculated using principal component analysis, i.e. the mean answers to narcissism, psychopathy, and Machiavellianism are multiplied with the respective factor loading and added up to one scalar. In column (2) the score on *psychopathy* is calculated by averaging the (standardized) answers to nine questions. The scores on *narcissism* and *Machiavellianism* are calculated analogously. *A*dditional controls include a dummy variable for the *gender* that is 1 for male individuals, dummy variables for *age* categories (with the age category 18-24 as the reference group), a dummy variable for the *country* (UK or Germany), dummy variables for *education* levels (with low education as a reference group), dummy variables for *income* (with being able to make ends meet with great difficulty as a reference group), a dummy variable for the *relationship status* that is 1 if the individual is single and lives alone and 0 if not, a dummy variable that indicates if the household includes *kids* under the age of 18, a dummy variable *risk group* that is 1 if the household includes individuals who belong to a risk group, i.e. elderly person(s) or someone with diagnosed chronic medical conditions such as heart or lung conditions or diabetes, self-reported *health risk attitude*, the variable *EQ-5D* that comprises the mean of (standardized) answers to five health related questions, and the variable *ICECAP* that comprises the mean of (standardized) answers to five questions on quality of life.

Table 11: Dark Triad personality and preventive behavior (full table 6)

	(1)<br>RE: Preventive Behavior	(2)<br>RE: Preventive Behavior
Dark Triad Score	0.009 (0.039)	
Psychopathy		-0.211*** (0.008)
Narcissism		0.115*** (0.015)
Machiavellianism		0.128 (0.100)
Health risk 1	-0.139** (0.063)	-0.137*** (0.050)
Health risk 2	-0.224*** (0.048)	-0.210*** (0.038)
Health risk 3	-0.349* (0.197)	-0.309 (0.201)
Health risk 4	-0.355** (0.180)	-0.313* (0.165)
Health risk 5	-0.414*** (0.157)	-0.350** (0.170)
Health risk 6	-0.442*** (0.020)	-0.376*** (0.024)
Health risk 7	-0.395** (0.164)	-0.336* (0.188)

Health risk 8	-0.341*** (0.127)	-0.274** (0.129)
Health risk 9	-0.257*** (0.057)	-0.203*** (0.061)
Health risk 10	-0.234*** (0.062)	-0.160*** (0.061)
No primary immunization	-0.313*** (0.040)	-0.296*** (0.049)
Boostered	0.116** (0.056)	0.129** (0.056)
Male	-0.147*** (0.005)	-0.121*** (0.008)
25-34	0.049*** (0.018)	0.048*** (0.016)
35-44	0.079*** (0.009)	0.064*** (0.021)
45-54	0.187*** (0.061)	0.159*** (0.041)
55-64	0.114 (0.171)	0.075 (0.128)
65+	0.062 (0.222)	0.006 (0.175)
Mid education	-0.074 (0.123)	-0.075 (0.116)
High education	-0.056 (0.047)	-0.064 (0.049)
Income (some diff)	-0.090*** (0.018)	-0.094*** (0.013)
Income (fairly easily)	-0.147*** (0.039)	-0.152*** (0.030)
Income (easily)	-0.171*** (0.060)	-0.183*** (0.054)
Single	-0.066*** (0.009)	-0.062*** (0.010)
Kids	-0.028 (0.049)	-0.019 (0.055)
Risk group	0.111 (0.089)	0.098 (0.088)
EQ-5D	0.054* (0.031)	0.033 (0.028)
ICECAP-A	0.092*** (0.018)	0.087*** (0.023)
cons	0.385** (0.160)	0.366** (0.144)
N	2,030	2,030

Heteroskedasticity-robust standard errors in parentheses, * $p < 0.10$, ** $p < 0.05$, *** $p < 0.01$.
Note: The table shows regression results using a random effects model with region random effects on country level. The dependent variable is a score on adherence to preventive behavior. Adherence to preventive behavior is assessed by averaging the (standardized) answers to six questions covering the following aspects: regular hand washing, covering nose and mouth when sneezing, physical distancing, avoiding shaking hand/hugging/kissing, using alcohol-based hand rub, and avoiding touching face. In column (1) the *Dark Triad score* is calculated using principal component analysis, i.e. the mean answers to narcissism, psychopathy, and Machiavellianism are multiplied with the respective factor loading and added up to one scalar. In column (2) the score on *psychopathy* is calculated by averaging the (standardized) answers to nine questions. The scores on *narcissism* and *Machiavellianism* are calculated analogously. Additional controls include a dummy variable for the *gender* that is 1 for male individuals, dummy variables for *age* categories (with the age category 18-24 as the reference group), dummy variables for *education* levels (with low education as a reference group), dummy variables for *income* (with being able to make ends meet with great difficulty as a reference group), a dummy variable for the *relationship status* that is 1 if the individual is single and lives alone and 0 if not, a dummy variable that indicates if the household includes *kids* under the age of 18, a dummy variable *risk group* that is 1 if the household includes individuals who belong to a risk group, i.e. elderly person(s) or someone with diagnosed chronic medical conditions such as heart or lung conditions or diabetes, self-reported *health risk attitude*, the variable *EQ-5D* that comprises the mean of (standardized) answers to five health related questions, the variable *ICECAP* that comprises the mean of (standardized) answers to five questions on quality of life, and the *vaccination status* (with having received primary immunization as a reference group).
