# Supplementary material for: Impact of Dark Triad personality traits on COVID-19 vaccination uptake and prevention efforts: insights from the European Covid Survey (ECOS)
Source: BMC Public Health. 2025 Apr 10;25:1352. doi: 10.1186/s12889-025-22471-3 (PMC11984049; doi:10.1186/s12889-025-22471-3)
Supplement: Supplementary file 3 — Supplementary Material 3: Appendix C. [file 12889_2025_22471_MOESM3_ESM.pdf]

Appendix C

This section shows sensitivity analyses. First, high correlations between the variables on Dark Triad personalities can result in inflated variances of the estimated coefficients and should be considered in the structure of the model. Our correlation analysis shows psychopathy with the highest intercorrelation to the other traits with a correlation of 0.55 and 0.52 to narcissism and Machiavellianism respectively. We find the weakest intercorrelation for narcissism and Machiavellianism (0.40). To test for multicollinearity we calculated the Variance Inflation Factor (VIF) but didn't obtain VIFs below the critical value. Nonetheless, we investigate the three personality traits narcissism, psychopathy, and Machiavellianism separately. The results on these specifications are shown in table 12 below with the coefficient estimates in column (1) being our baseline model from table 4 column (2). The decrease in the coefficient estimate on psychopathy can be attributed to the relatively high correlation to the other Dark Triad personality constructs. Nevertheless, we find our main results to be largely unaffected with significant estimates on narcissism only.

Table 12: Booster uptake for each Dark Triad personality trait separately

	(1)<br>Logit: Booster	(2)<br>Logit: Booster	(3)<br>Logit: Booster	(4)<br>Logit: Booster
Psychopathy	0.142 (0.128)	0.016 (0.108)		
Narcissism	-0.276** (0.138)		-0.215* (0.121)	
Machiavellianism	-0.028 (0.114)			-0.048 (0.102)
Additional controls	yes	yes	yes	yes
N	1,740	1,740	1,740	1,740
Pseudo R^2	0.176	0.174	0.175	0.174

Note: Significance level indicate the following: * $p < 0.10$, ** $p < 0.05$, *** $p < 0.01$. Standard errors are shown in parentheses. The table shows regression results using a logit model. The dependent variable is the vaccination status, e.g. having received a booster shot (when having received primary immunization). The dependent variable is 1 if the individual has received a booster shot and 0 if the individual has received primary immunization but no booster shot. In column (1) the score on *psychopathy* is calculated by averaging the (standardized) answers to nine questions. The scores on *narcissism* and *Machiavellianism* are calculated analogously. In column (2), (3), and (4) regression results investigating *psychopathy*, *narcissism*, and *Machiavellianism* separately are shown respectively. *A*dditional controls include a dummy variable for the *gender* that is 1 for male individuals, dummy variables for *age* categories (with the age category 18-24 as the reference group), a dummy variable for the *country* (UK or Germany), dummy variables for *education* levels (with low education as a reference group), dummy variables for *income* (with being able to make ends meet with great difficulty as a reference group), a dummy variable for the *relationship status* that is 1 if the individual is single and lives alone and 0 if not, a dummy variable that indicates if the household includes *kids* under the age of 18, a dummy variable *risk group* that is 1 if the household includes individuals who belong to a risk group, i.e. elderly person(s) or someone with diagnosed chronic medical conditions such as heart or lung conditions or diabetes, self-reported *health risk attitude*, the variable *EQ-5D* that comprises the mean of (standardized) answers to five health related questions, and the variable *ICECAP* that comprises the mean of (standardized) answers to five questions on quality of life.

The respective results with regard to primary immunization (table 13) and simple preventive behavior (table 14) where we investigate the three personality traits separately are shown below.

Table 13: Primary immunization for each Dark Triad personality trait separately

	(1)<br>Logit: Prim Vacc	(2)<br>Logit: Prim Vacc	(3)<br>Logit: Prim Vacc	(4)<br>Logit: Prim Vacc
Psychopathy	-0.299** (0.146)	-0.317*** (0.124)		
Narcissism	-0.077 (0.158)		-0.215 (0.137)	
Machiavellianism	0.030 (0.129)			-0.108 (0.114)
Additional controls	yes	yes	yes	yes
N	2,030	2,030	2,030	2,030
Pseudo R^2	0.105	0.105	0.102	0.101

Note: Significance level indicate the following: * $p < 0.10$, ** $p < 0.05$, *** $p < 0.01$. Standard errors are shown in parentheses. The table shows regression results using a logit model. The dependent variable is the vaccination status, e.g. having received primary immunization. The dependent variable is 1 if the individual has received primary immunization as having received two or three shots and 0 otherwise. In column (1) the score on *psychopathy* is calculated by averaging the (standardized) answers to nine questions. The scores on *narcissism* and *Machiavellianism* are calculated analogously. In column (2), (3), and (4) regression results investigating *psychopathy*, *narcissism*, and *Machiavellianism* separately are shown respectively. *A*dditional controls include a dummy variable for the *gender* that is 1 for male individuals, dummy variables for *age* categories (with the age category 18-24 as the reference group), a dummy variable for the *country* (UK or Germany), dummy variables for *education* levels (with low education as a reference group), dummy variables for *income* (with being able to make ends meet with great difficulty as a reference group), a dummy variable for the *relationship status* that is 1 if the individual is single and lives alone and 0 if not, a dummy variable that indicates if the household includes *kids* under the age of 18, a dummy variable *risk group* that is 1 if the household includes individuals who belong to a risk group, i.e. elderly person(s) or someone with diagnosed chronic medical conditions such as heart or lung conditions or diabetes, self-reported *health risk attitude*, the variable *EQ-5D* that comprises the mean of (standardized) answers to five health related questions, and the variable *ICECAP* that comprises the mean of (standardized) answers to five questions on quality of life.

Table 14: Preventive behavior for each Dark Triad personality trait separately

	(1) RE: Prev Beh	(2) RE: Prev Beh	(3) RE: Prev Beh	(4) RE: Prev Beh
Psychopathy	-0.211*** (0.008)	-0.105** (0.044)		
Narcissism	0.115*** (0.015)		0.065 (0.051)	
Machiavellianism	0.128 (0.100)			0.075 (0.096)
Additional controls	yes	yes	yes	yes
N	2,030	2,030	2,030	2,030

Heteroskedasticity-robust standard errors in parentheses, * $p < 0.10$, ** $p < 0.05$, *** $p < 0.01$.
Note: The table shows regression results using a random effects model with region random effects on country level. The dependent variable is a score on adherence to preventive behavior. Adherence to preventive behavior is assessed by averaging the (standardized) answers to six questions covering the following aspects: regular hand washing, covering nose and mouth when sneezing, physical distancing, avoiding shaking hand/hugging/kissing, using alcohol-based hand rub, and avoiding touching face. In column (1) the score on *psychopathy* is calculated by averaging the (standardized) answers to nine questions. The scores on *narcissism* and *Machiavellianism* are calculated analogously. In column (2), (3), and (4) regression results investigating *psychopathy*, *narcissism*, and *Machiavellianism* separately are shown respectively. *A*dditional controls include a dummy variable for the *gender* that is 1 for male individuals, dummy variables for *age* categories (with the age category 18-24 as the reference group), dummy variables for *education* levels (with low education as a reference group), dummy variables for *income* (with being able to make ends meet with great difficulty as a reference group), a dummy variable for the *relationship status* that is 1 if the individual is single and lives alone and 0 if not, a dummy variable that indicates if the household includes *kids* under the age of 18, a dummy variable *risk group* that is 1 if the household includes individuals who belong to a risk group, i.e. elderly person(s) or someone with diagnosed chronic medical conditions such as heart or lung conditions or diabetes, self-reported *health risk attitude*, the variable *EQ-5D* that comprises the mean of (standardized) answers to five health related questions, the variable *ICECAP* that comprises the mean of (standardized) answers to five questions on quality of life, and the *vaccination status* (with having received primary immunization as a reference group).

Second, we exclude participants from the analysis (N=150) who always pick the middle answer on the 5-point Likert-scale, i. e. "neither agree nor disagree (3)", with regard to all of the nine statements per trait on at least one of the Dark Triad personalities to mitigate potentially unreliable reporting. One potential shortfall of the 5-point Likert-scale could be that the answer in the middle of the scale can serve as an unconscious anchor. Participants can insufficiently adjust this anchor because they follow a simple (unconscious) heuristic or simply because of unmotivated reporting and inertia. To alleviate concerns with regard to potentially biased reporting, we rerun our regressions with a reduced sample. Our results of this additional robustness check are depicted in table 15, with the estimates shown in columns (1) and (2) being our baseline models from table 4. Columns (3) and (4) of table 15 show estimated coefficients on the restricted sample. Again, we find highly consistent estimates and robust significance levels.

Table 15: Dark Triad personality and booster uptake accounting for (potential) reporting bias

	(1) Logit: Booster	(2) Logit: Booster	(3) Logit: Booster	(4) Logit: Booster
Dark Triad Score	-0.042 (0.050)		-0.048 (0.053)	
Psychopathy		0.142 (0.128)		0.118 (0.142)
Narcissism		-0.276** (0.138)		-0.285** (0.145)

Machiavellianism		-0.028 (0.114)		-0.016 (0.123)
Additional controls	yes	yes	yes	yes
N	1,740	1,740	1,590	1,590
Pseudo R^2	0.174	0.176	0.181	0.182

Note: Significance level indicate the following: * $p < 0.10$, ** $p < 0.05$, *** $p < 0.01$. Standard errors are shown in parentheses. The table shows regression results using a logit model. The dependent variable is the vaccination status, e.g. having received a booster shot (when having received primary immunization). The dependent variable is 1 if the individual has received a booster shot and 0 if the individual has received primary immunization but no booster shot. In column (1) the *Dark Triad score* is calculated using principal component analysis, i.e. the mean answers to narcissism, psychopathy, and Machiavellianism are multiplied with the respective factor loading and added up to one scalar. In column (2) the score on *psychopathy* is calculated by averaging the (standardized) answers to nine questions. The scores on *narcissism* and *Machiavellianism* are calculated analogously. Columns (3) and (4) show regression results for our restricted sample investigating the *Dark Triad score* and each Dark Triad personality traits separately, respectively. We exclude participants from the analysis (N=150) who always pick the middle answer on the 5-point Likert-scale, i. e. "neither agree nor disagree (3)", with regard to the statements on at least one of the Dark Triad personalities. *A*dditional controls include a dummy variable for the *gender* that is 1 for male individuals, dummy variables for *age* categories (with the age category 18-24 as the reference group), a dummy variable for the *country* (UK or Germany), dummy variables for *education* levels (with low education as a reference group), dummy variables for *income* (with being able to make ends meet with great difficulty as a reference group), a dummy variable for the *relationship status* that is 1 if the individual is single and lives alone and 0 if not, a dummy variable that indicates if the household includes *kids* under the age of 18, a dummy variable *risk group* that is 1 if the household includes individuals who belong to a risk group, i.e. elderly person(s) or someone with diagnosed chronic medical conditions such as heart or lung conditions or diabetes, self-reported *health risk attitude*, the variable *EQ-5D* that comprises the mean of (standardized) answers to five health related questions, and the variable *ICECAP* that comprises the mean of (standardized) answers to five questions on quality of life.

Again, table 16 and table 17 below show the results using the reduced sample for primary immunization and preventive behavior, respectively.

Table 16: Dark Triad personality and primary immunization accounting for (potential) reporting bias

	(1) Logit: Prim Vacc	(2) Logit: Prim Vacc	(3) Logit: Prim Vacc	(4) Logit: Prim Vacc
Dark Triad Score	-0.116** (0.056)		-0.123** (0.061)	
Psychopathy		-0.299** (0.146)		-0.328** (0.162)
Narcissism		-0.077 (0.158)		-0.046 (0.168)
Machiavellianism		0.030 (0.129)		0.004 (0.140)
Additional controls	yes	yes	yes	yes
N	2,030	2,030	1,840	1,840
Pseudo R^2	0.103	0.105	0.105	0.106

Note: Significance level indicate the following: * $p < 0.10$, ** $p < 0.05$, *** $p < 0.01$. Standard errors are shown in parentheses. The table shows regression results using a logit model. The dependent variable is the vaccination status, e.g. having received primary immunization. The dependent variable is 1 if the individual has received primary immunization as having received two or three shots and 0 otherwise. In column (1) the *Dark Triad score* is calculated using principal component analysis, i.e. the mean answers to narcissism, psychopathy, and Machiavellianism are multiplied with the respective factor loading and added up to one scalar. In column (2) the score on *psychopathy* is calculated by averaging the (standardized) answers to nine questions. The scores on *narcissism* and *Machiavellianism* are calculated analogously. Columns (3) and (4) show regression results for our restricted sample investigating the *Dark Triad score* and each Dark Triad personality traits separately, respectively. We exclude participants from the analysis (N=190) who always pick the middle answer on the 5-point Likert-scale, i. e. "neither agree nor disagree (3)", with regard to the statements on at least one of the Dark Triad personalities. *A*dditional controls include a dummy variable for the *gender* that is 1 for male individuals, dummy variables for *age* categories (with the age category 18-24 as the reference group), a dummy variable for the *country* (UK or Germany), dummy variables for *education* levels (with low education as a reference group), dummy variables for *income* (with being able to make ends meet with great difficulty as a reference group), a dummy variable for the *relationship status* that is 1 if the individual is single and lives alone and 0 if not, a dummy variable that indicates if the household includes *kids* under the age of 18, a dummy variable *risk group* that is 1 if the household includes individuals who belong to a risk group, i.e. elderly person(s) or someone with diagnosed chronic medical conditions such as heart or lung conditions or diabetes, self-reported *health risk attitude*, the variable *EQ-5D* that comprises the mean of (standardized) answers to five health related questions, and the variable *ICECAP* that comprises the mean of (standardized) answers to five questions on quality of life.

Table 17: Dark Triad personality and preventive behavior accounting for (potential) reporting bias

	(1)<br>RE: Prev Beh	(2)<br>RE: Prev Beh	(3)<br>RE: Prev Beh	(4)<br>RE: Prev Beh
Dark Triad Score	0.009 (0.039)		0.008 (0.044)	
Psychopathy		-0.211*** (0.008)		-0.231*** (0.009)
Narcissism		0.115*** (0.015)		0.120*** (0.016)
Machiavellianism		0.128 (0.100)		0.129 (0.106)
Additional controls	yes	yes	yes	yes
N	2,030	2,030	1,840	1,840

Heteroskedasticity-robust standard errors in parentheses, * $p < 0.10$, ** $p < 0.05$, *** $p < 0.01$.
Note: The table shows regression results using a random effects model with region random effects on country level. The dependent variable is a score on adherence to preventive behavior. Adherence to preventive behavior is assessed by averaging the (standardized) answers to six questions covering the following aspects: regular hand washing, covering nose and mouth when sneezing, physical distancing, avoiding shaking hand/hugging/kissing, using alcohol-based hand rub, and avoiding touching face. In column (1) the *Dark Triad score* is calculated using principal component analysis, i.e. the mean answers to narcissism, psychopathy, and Machiavellianism are multiplied with the respective factor loading and added up to one scalar. In column (2) the score on *psychopathy* is calculated by averaging the (standardized) answers to nine questions. The scores on *narcissism* and *Machiavellianism* are calculated analogously. Columns (3) and (4) show regression results for our restricted sample investigating the *Dark Triad score* and each Dark Triad personality traits separately, respectively. We exclude participants from the analysis (N=190) who always pick the middle answer on the 5-point Likert-scale, i. e. "neither agree nor disagree (3)", with regard to the statements on at least one of the Dark Triad personalities. *A*dditional controls include a dummy variable for the *gender* that is 1 for male individuals, dummy variables for *age* categories (with the age category 18-24 as the reference group), dummy variables for *education* levels (with low education as a reference group), dummy variables for *income* (with being able to make ends meet with great difficulty as a reference group), a dummy variable for the *relationship status* that is 1 if the individual is single and lives alone and 0 if not, a dummy variable that indicates if the household includes *kids* under the age of 18, a dummy variable *risk group* that is 1 if the household includes individuals who belong to a risk group, i.e. elderly person(s) or someone with diagnosed chronic medical conditions such as heart or lung conditions or diabetes, self-reported *health risk attitude*, the variable *EQ-5D* that comprises the mean of (standardized) answers to five health related questions, the variable *ICECAP* that comprises the mean of (standardized) answers to five questions on quality of life, and the *vaccination status* (with having received primary immunization as a reference group).

So far, we assumed that the error term in our model is distributed according to a logistic distribution. As an additional robustness check we estimate probit models and do not find significantly different results (table 18). Again, our baseline models from table 4 are shown in columns (1) and (2). Columns (3) and (4) of table 18 show the estimated probit models.

Table 18: Dark Triad personality and booster uptake (logit versus probit)

	(1)<br>Logit: Booster	(2)<br>Logit: Booster	(3)<br>Probit: Booster	(4)<br>Probit: Booster
Dark Triad Score	-0.042 (0.050)		-0.024 (0.029)	
Psychopathy		0.142 (0.128)		0.068 (0.075)
Narcissism		-0.276** (0.138)		-0.148* (0.081)
Machiavellianism		-0.028 (0.114)		-0.011 (0.068)
Additional controls	yes	yes	yes	yes
N	1,740	1,740	1,740	1,740
Pseudo R^2	0.174	0.176	0.177	0.178

Note: Significance level indicate the following: * $p < 0.10$, ** $p < 0.05$, *** $p < 0.01$. Standard errors are shown in parentheses. The table shows regression results using a logit model (columns (1) and (2)) and a probit model (columns (3) and (4)). The dependent variable is the vaccination status, e.g. having received a booster shot (when having received primary immunization). The dependent variable is 1 if the individual has received a booster shot and 0 if the individual has received primary immunization but no booster shot. In column (1) and column (3) the *Dark Triad score* is calculated using principal component analysis, i.e. the mean answers to narcissism, psychopathy, and Machiavellianism are multiplied with the respective factor loading and added up to one scalar. In column (2) and column (4) the score on *psychopathy* is calculated by averaging the (standardized) answers to nine questions. The scores on *narcissism* and *Machiavellianism* are calculated analogously. Additional controls include a dummy variable for the *gender* that is 1 for male individuals, dummy variables for *age* categories (with the age category 18-24 as the reference group), a dummy variable for the *country* (UK or Germany), dummy variables for *education* levels (with low education as a reference group), dummy variables for *income* (with being able to make ends meet with great difficulty as a reference group), a dummy variable for the *relationship status* that is 1 if the individual is single and lives alone and 0 if not, a dummy variable that indicates if the household includes *kids* under the age of 18, a dummy variable *risk group* that is 1 if the household includes individuals who belong to a risk group, i.e. elderly person(s) or someone with diagnosed chronic medical conditions such as heart or lung conditions or diabetes, self-reported *health risk attitude*, the variable *EQ-5D* that comprises the mean of (standardized) answers to five health related questions, and the variable *ICECAP* that comprises the mean of (standardized) answers to five questions on quality of life.

Table 19 shows regression results with regard to primary immunization using a probit model.

Table 19: Dark Triad personality and primary immunization (logit versus probit)

	(1)<br>Logit: Prim Vacc	(2)<br>Logit: Prim Vacc	(3)<br>Probit: Prim Vacc	(4)<br>Probit: Prim Vacc
Dark Triad Score	-0.116** (0.056)		-0.065** (0.031)	
Psychopathy		-0.299** (0.146)		-0.166** (0.080)
Narcissism		-0.077 (0.158)		-0.051 (0.085)
Machiavellianism		0.030 (0.129)		0.021 (0.069)
Additional controls	yes	yes	yes	yes
N	2,030	2,030	2,030	2,030
Pseudo R^2	0.103	0.105	0.105	0.107

Note: Significance level indicate the following: * $p < 0.10$, ** $p < 0.05$, *** $p < 0.01$. Standard errors are shown in parentheses. The table shows regression results using a logit model (columns (1) and (2)) and a probit model (columns (3) and (4)). The dependent variable is the vaccination status, e.g. having received primary immunization. The dependent variable is 1 if the individual has received primary immunization as having received two or three shots and 0 otherwise. In column (1) and column (3) the *Dark Triad score* is calculated using principal component analysis, i.e. the mean answers to narcissism, psychopathy, and Machiavellianism are multiplied with the respective factor loading and added up to one scalar. In column (2) and column (4) the score on *psychopathy* is calculated by averaging the (standardized) answers to nine questions. The scores on *narcissism* and *Machiavellianism* are calculated analogously. *A*dditional controls include a dummy variable for the *gender* that is 1 for male individuals, dummy variables for *age* categories (with the age category 18-24 as the reference group), a dummy variable for the *country* (UK or Germany), dummy variables for *education* levels (with low education as a reference group), dummy variables for *income* (with being able to make ends meet with great difficulty as a reference group), a dummy variable for the *relationship status* that is 1 if the individual is single and lives alone and 0 if not, a dummy variable that indicates if the household includes *kids* under the age of 18, a dummy variable *risk group* that is 1 if the household includes individuals who belong to a risk group, i.e. elderly person(s) or someone with diagnosed chronic medical conditions such as heart or lung conditions or diabetes, self-reported *health risk attitude*, the variable *EQ-5D* that comprises the mean of (standardized) answers to five health related questions, and the variable *ICECAP* that comprises the mean of (standardized) answers to five questions on quality of life.

Also note that we use the mean of the answers on all 27 Dark Triad items as a Dark Triad score instead of the Dark Triad score derived by Principal Component Analysis as well as simple unstandardized scores for each personality trait. Summary statistics are shown in table 20. We find extremely robust coefficient estimates and significance levels (see table 21 and table 22).

Table 20: Summary statistics: Dark Triad personality (unstandardized scores)

	Mean	Median	Min	Max	SD
Dark Triad score	2.65	2.67	1.19	4.26	0.56
Narcissism	2.65	2.78	1.00	4.33	0.60
Machiavellianism	3.04	3.00	1.00	5.00	0.68
Psychopathy	2.25	2.22	1.00	4.22	0.78
Observations	2,030				

Note: The table provides a descriptive summary (mean, median, minimum, maximum, and standard deviation) of the Dark Triad personality in our sample. The *Dark Triad score* is the average of the (unstandardized) answers to 27 items while *narcissism, Machiavellianism*, and *psychopathy* are the average scores of (unstandardized) answers to nine questions per personality trait.

Table 21: Dark Triad personality and primary immunization using "simple" DT scores

	(1)<br>Logit: Primary Vaccination	(2)<br>Logit: Primary Vaccination
Dark Triad Score	-0.296** (0.142)	
Psychopathy		-0.251** (0.127)
Narcissism		-0.084 (0.147)
Machiavellianism		0.047 (0.120)

Additional controls	yes	yes
N	2,030	2,030
Pseudo R^2	0.103	0.105

Note: Note: Significance level indicate the following: * $p < 0.10$, ** $p < 0.05$, *** $p < 0.01$. Standard errors are shown in parentheses. The table shows regression results using a logit model. The dependent variable is the vaccination status, e.g. having received primary immunization. The dependent variable is 1 if the individual has received primary immunization as having received two or three shots and 0 otherwise. In column (1) the *Dark Triad score* is calculated by taking the mean of all answers to the 27 items covering narcissism, psychopathy, and Machiavellianism. In column (2) the score on *psychopathy* is calculated by averaging the (unstandardized) answers to nine questions. The scores on *narcissism* and *Machiavellianism* are calculated analogously. *A*dditional controls include a dummy variable for the *gender* that is 1 for male individuals, dummy variables for *age* categories (with the age category 18-24 as the reference group), a dummy variable for the *country* (UK or Germany), dummy variables for *education* levels (with low education as a reference group), dummy variables for *income* (with being able to make ends meet with great difficulty as a reference group), a dummy variable for the *relationship status* that is 1 if the individual is single and lives alone and 0 if not, a dummy variable that indicates if the household includes *kids* under the age of 18, a dummy variable *risk group* that is 1 if the household includes individuals who belong to a risk group, i.e. elderly person(s) or someone with diagnosed chronic medical conditions such as heart or lung conditions or diabetes, self-reported *health risk attitude*, the variable *EQ-5D* that comprises the mean of (standardized) answers to five health related questions, and the variable *ICECAP* that comprises the mean of (standardized) answers to five questions on quality of life.

Table 22: Dark Triad personality and booster uptake using "simple" DT scores

	(1) Logit: Booster	(2) Logit: Booster
Dark Triad Score	-0.098 (0.126)	
Psychopathy		0.122 (0.113)
Narcissism		-0.272** (0.130)
Machiavellianism		-0.008 (0.108)
Additional controls	yes	yes
N	1,740	1,740
Pseudo R^2	0.174	0.176

Note: Note: Significance level indicate the following: * $p < 0.10$, ** $p < 0.05$, *** $p < 0.01$. Standard errors are shown in parentheses. The table shows regression results using a logit model. The dependent variable is the vaccination status, e.g. having received a booster shot (when having received primary immunization). The dependent variable is 1 if the individual has received a booster shot and 0 if the individual has received primary immunization but no booster shot. In column (1) the *Dark Triad score* is calculated by taking the mean of all answers to the 27 items covering narcissism, psychopathy, and Machiavellianism. In column (2) the score on *psychopathy* is calculated by averaging the (unstandardized) answers to nine questions. The scores on *narcissism* and *Machiavellianism* are calculated analogously. *A*dditional controls include a dummy variable for the *gender* that is 1 for male individuals, dummy variables for *age* categories (with the age category 18-24 as the reference group), a dummy variable for the *country* (UK or Germany), dummy variables for *education* levels (with low education as a reference group), dummy variables for *income* (with being able to make ends meet with great difficulty as a reference group), a dummy variable for the *relationship status* that is 1 if the individual is single and lives alone and 0 if not, a dummy variable that indicates if the household includes *kids* under the age of 18, a dummy variable *risk group* that is 1 if the household includes individuals who belong to a risk group, i.e. elderly person(s) or someone with diagnosed chronic medical conditions such as heart or lung conditions or diabetes, self-reported *health risk attitude*, the variable *EQ-5D* that comprises the mean of (standardized) answers to five health related questions, and the variable *ICECAP* that comprises the mean of (standardized) answers to five questions on quality of life.
